# Hazelnut as Ingredient in Dairy Sheep Diet: Effect on Sensory and Volatile Profile of Cheese

**DOI:** 10.3389/fnut.2019.00125

**Published:** 2019-08-08

**Authors:** Margherita Caccamo, Bernardo Valenti, Giuseppe Luciano, Alessandro Priolo, Teresa Rapisarda, Giovanni Belvedere, Vita Maria Marino, Sonia Esposto, Agnese Taticchi, Maurizio Servili, Mariano Pauselli

**Affiliations:** ^1^Consorzio per la Ricerca nel Settore della Filiera Lattiero-Casearia e dell'Agroalimentare, Ragusa, Italy; ^2^Dipartimento di Scienze Agrarie, Alimentari e Ambientali, University of Perugia, Perugia, Italy; ^3^Dipartimento di Agricoltura, Alimentazione e Ambiente, University of Catania, Catania, Italy

**Keywords:** dairy sheep, hazelnut, sensory properties, diet supplement, sheep cheese

## Abstract

The opportunity of replacing expensive feedstuffs with agro-industrial by-products in the diet of food producing animals is raising increasing interest while addressing global concern for the scarcity of natural resources and environmental impact of livestock farming. Hazelnut peels, rich in fiber and vitamins and characterized by a high concentration of fats, is considered a suitable ingredient to be included in the diet of ruminants. The aim of this research was to assess the effect of dietary hazelnut peels on the chemical and sensory properties of sheep cheese during refrigerated storage. To this purpose, 20 Comisana lactating ewes were randomly assigned to two experimental groups, control (C) and hazelnut peels (HP), balanced for parity, milk yield and body weight. Bulk milk collected from the 2 groups was used to produce 5 Pecorino cheeses for each group. After 40 d of aging, each cheese of each experimental group was divided into 3 pieces: 1 piece was sampled for analyses (C0, HP0) and 2 were wrapped in PVC film, simulating the condition of pre-wrapped products, and analyzed after 7 (C7, HP7) and 14 days of storage (C14, HP14) at 8°C with 80% moisture. The cheeses were analyzed for chemical and fatty acid composition, sensory analysis, odor active compounds and SmartNose. As expected, HP cheeses presented a higher lipid content compared to C, a lower content in SFA and PUFA, and a greater content in MUFA. A triangle test revealed a clear distinction between the 2 groups (α = 0.01) The sensory profile showed a significant effect on holes (*P* < 0.05) and a marginal production of off-flavors linked to spicy and acid attributes for HP cheeses The volatile profile of C and HP cheese samples showed a good similarity, partially explained by the short ripening time and the absence of 2-nonanone in HP7, suggesting a higher antioxidant protection grade of this cheese compared to the others. These results were confirmed by Smart Nose analysis. Further studies on vitamin content should be conducted in order to investigate the interactions between the presence of antioxidant volatile compounds and the oxidative stability of ewe cheese.

## Introduction

The global concern for the scarcity of natural resources dramatically increased over the last decades. For this reason, FAO and EU promote the principles of 3R (Reduce, Reuse, and Recycle) for a sustainable development in all the productive sectors, including livestock production. Animal feedstuffs production, processing and transport are highly demanding in terms of natural resources. Moreover, livestock farming greatly impacts on the environment. In this context, the inclusion of agro-industrial by-products in the diet of food producing animals as a replacer of conventional, and more expensive feedstuffs, is under investigation to mitigate the impact of livestock production (1). Interestingly, most of the food-derived by-products are rich of bioactive molecules that can exert positive effects both on animal welfare and product quality (2).

Hazelnut (*Coryllus Avellana* L.) is worldwide consumed and the global production accounts for more than 1 million tons per year. Hazelnut can be consumed as such, but the biggest part of the production is destined to pastry and chocolate industry. Hazelnut peel (HP) is the by-product that results from the roasting phase during the industrial processing of the hazelnut and represents about 2.5% of total hazelnut kernel weight (3). Indeed, hazelnut peel is the perisperm of the kernel and need to be removed from the fruit to prevent food off-flavors and off-colors due to the great content of phenolic compounds (4).

Thanks to its chemical composition, HP could be considered a suitable ingredient to be included in the diet of ruminants. In particular, HP is rich in fiber (~30%), fat (~20%) and even at lower percentage can afford protein (~7%) (5). The oil fraction is characterized by the high content of unsaturated fatty acids (UFA; principally oleic and linoleic acid). Also, HP is rich of vitamins, mostly represented by vitamin E. Lastly, HP is a natural source of polyphenols. Specifically, del Rio et al. (6) report that among the total phenolic compounds (675 mg/100 g DM) gallic acid, procyanidin dimers and trimers, flavan-3-ols, flavonols and hydrolizable tannins as glansreginin A, B, and C represent the main part.

The bioactive molecules contained in the HP, or their derivatives, could be transferred to ruminant products affecting their nutritive value and sensory properties (7). Indeed, it is well-accepted that dietary unsaturated fatty acids can be used to improve the healthiness of meat, milk and cheese (8). However, increasing the proportion of lipids highly sensitive to oxidation can play a role in the development of the aroma in repined products such as cheese (9). Also, vitamins and phenolic compounds, besides adding value to the healthy properties of ruminant products, could act as antioxidant (or modulators of the bacterial activity) thus influencing the maturation processes of the fermented products (10).

In the literature, the use of dietary plant extracts rich in polyphenols, such as condensed or hydrolysable tannins, has been investigated to improve quality traits of ruminant products (7, 11). However, the use of plant extract could increase the cost of the animal diet. In the literature the effect of the inclusion of hazelnut peel in animal diet on the quality of ruminant products is lacking. In this context, recycling hazelnut peel as a source of bioactive substances for feeding livestock could represent an innovation in the field of research in animal production. Additionally, the chemical composition of hazelnut peel may justify a high level of inclusion in the animal diet, which would allow a more effective exploitation of this by-product as a replacer of traditionally used feedstuffs.

Considering the all above, the aim of this research was to assess, for the first time, the effect of dietary hazelnut peel on the chemical and the sensory properties of sheep cheese during refrigerated storage.

## Materials and Methods

### Animals and Diets

The trial was carried out at the Experimental Farm of the Department of Agricultural, Food and Environmental Science of the University of Perugia. Twenty multiparous Comisana lactating ewes with 89 ± 10 days in milk were randomly assigned to two experimental groups (*n* = 10), namely control (C) and hazelnut (HP), balanced for parity (2.3 vs. 2.5), current milk yield (824 vs. 804 g/d) and BW (62.6 vs. 64.0 kg). The animals were kept in two separate boxes (one per group) with sawdust bedding. The experimental trial lasted 28 days after 14-day adaptation period during which the animals were gradually adapted to the respective diet composed by chopped alfalfa hay offered *ad libitum* (particle size >4 cm in length) and 800 g/ewe/day of a pelleted concentrate containing 370 g/kg DM of dried beet pulp (C) or 360 g/kg DM of hazelnut peels (HP). The concentrate, formulated to cover the nutrition requirements of a sheep weighing 68 kg producing 1 kg milk per day with 6.5% fat (12), was given individually at the morning and afternoon milking (400 g DM at each milking) until complete consumption.

Representative samples of the offered feeds were analyzed for neutral detergent fiber (NDF) according to Van Soest et al. (13). Furthermore, crude protein, crude fat (ether extract) and ash were analyzed according to methods 976.06, 920.39, and 942.05, respectively (14). In addition, lipids from the individual feeds were extracted and converted to fatty acid methyl esters (FAME) with a 1-step procedure using chloroform (15) and 2% (v/v) sulfuric acid in methanol (16) to determine the fatty acid profile. Non-adecanoic acid was used as an internal standard. Gas chromatographic analysis was carried out as later described for the analysis of cheese fatty acids. Table 1 reports the ingredients and the chemical composition of the offered feeds.

**Table 1:** Ingredients and chemical composition of diets used in the experiment including concentrates containing either beet pulp (C) or hazelnut peel (HP).

			**Experimental concentrates**	
	**Hay**	**Hazelnut peel**	**C**	**HP**
**INGREDIENTS (G/KG DRY MATTER)**				
Hazelnut peel			-	360.2
Barley			345.1	329.5
Wheat bran			98.6	97.0
Soybean meal			140.8	168.2
Dried beet pulp			369.8	-
Molasses			25.4	25.0
Calcium carbonate			5.0	5.0
Sodium bicarbonate			5.0	5.0
Dicalcium phosphate			5.0	5.0
Sodium chloride			5.0	5.0
**CHEMICAL COMPOSITION (G/KG DRY MATTER)**				
Crude protein	149.9	78.6	157.7	162.7
Ether extract	15.8	226.3	16.3	91.5
NDF	527.7	510.7	302.2	358.3
ADF	429.3	387.7	135.4	225.8
ADL	95.7	202.8	15	75.6
Ash	75.6	24.8	63.9	51.8
**PROTEIN FRACTIONS**				
A	39.1	1.8	21.5	8.2
B1	6.9	3.7	5.9	19.0
B2	71.0	18.1	100.0	73.1
B3	19.3	1.6	24.1	32.7
C	13.7	53.3	6.1	29.7
**FATTY ACIDS (G/100G DM)**				
14:0	0,007	0,015	0,004	0,007
16:0	0,193	1,041	0,374	0,663
18:0	0,039	0,383	0,033	0,17
cis-9 18:1	0,044	11,067	0,268	4,485
cis-9 cis-12 18:2	0,145	2,018	0,883	1,497
cis-9 cis-12 cis-15 18:3	0.245	0.031	0.087	0.078

The research activity reported in this paper treated the supplementation fed to animals by including either beet pulp or hazelnut peel in the concentrate. Therefore, this project is not regulated by the Directive 2010/63/EU art. 1, point 4, letter f, on the protection of animals used for scientific purposes, according to which the directive does not apply to the practices not likely to cause pain, suffering, distress or prolonged damage equivalent or superior to that caused by the insertion of a needle according to the good veterinary practices. The feeding trial followed the ordinary practices of dairy sheep farms. Therefore, approval was not needed according to institutional and national guidelines. Nevertheless, all the experimental procedures adopted agree with the European Union guidelines about experimental animals (Gazzetta Ufficiale 61, 2004).

### Cheese Production and Shelf-Life Evaluation

Bulk milk from each of the two experimental groups was collected during last week of the experimental period and stored at −30°C until the quantity of 40 kg was reached. The milk was thawed in cold condition at 5°C. The cheese-making procedure was performed according to a traditional technique reported by Mughetti et al. (17), with modifications. Briefly, the milk was heated at 39°C and a mixed-strain starter culture (MW039S SACCO) was added and incubated for 10 min. Then, a liquid calf-lamb rennet was added (22g/100 L). After 20 min the curd was turned on the surface and broken in 3cm large square to let the whey bleed. After 5 min the curd was further broken until the corn grain dimension was reached. Finally, the curd was put on plastic basket and the whey was left bleeding until the pH reached 5.5 level. A total of 10 cheeses were produced (5 per experimental group). After 24 h refrigeration at 7°C, all the cheeses were put in brine for 12 h and aged in a cold room for 40 days.

After the aging, each cheese was divided into 3 pieces, each representing a subsample. One of the three subsamples of each cheese was destined to the analyses described below without storage (C0 and HP0). The other two subsamples were wrapped using a PVC film, simulating the method adopted in the stores for the pre-wrapped products, and analyzed after 7 (C7, HP7) or 14 days of storage (C14, HP14) at 8°C with 80% moisture.

## Cheese Analyses

### Chemical and Fatty Acid Composition

Cheese samples were analyzed to determine moisture according to the method proposed by Bradley and Vanderwarn (18), lipid content according to the Gerber - Van Gulik method (ISO 1975) and protein content (total nitrogen x 6.38) determined using the Kjeldhal method. Cheese fatty acid composition was determined by gas-chromatography. Fat was extracted from 5 g of finely minced cheese using a mixture of chloroform and methanol (2:1, v/v) as described by Folch et al. (19) and 30 mg of lipids were converted to FA methylesters (FAME) by base catalyzed transesterification (20) using 0.5 mL of sodium methoxide in methanol 0.5 N and 1 mL of hexane containing 19:0 as an internal standard. Gas chromatographic analysis was performed on a Trace Thermo Finnigan GC system (ThermoQuest, Milan, Italy) equipped with a flame-ionization detector and a 100 m fused silica capillary column (0.25 mm i.d., 0.25-μm film thickness; SP-24056; Supelco Inc., Bellefonte, PA) and helium as the carrier gas (1 mL/min). FAME profile in a 1-μL sample volume (split ratio 1:80) was determined according to the temperature gradient program described by Valenti et al. (21). The oven temperature was programmed at 50°C and held for 4 min, then increased to 120°C at 10°C/min, held for 1 min, then increased up to 180°C at 5°C/min, held for 18 min, then increased up to 200°C at 2°C/min, held for 15 min, and then increased up to 230°C at 2°C/min, held for 19 min. The injector and detector temperatures were at 270 and 300°C, respectively. FAME identification was based on a commercial mixture of standard FAME (Nu-Chek Prep Inc., Elysian, MN, USA), individual standard FAME (Larodan Fine Chemicals, Malmo, Sweden). Fatty acids were expressed as percentage of total fatty acids.

### Sensory Analysis

The cheese for the groups C and HP were evaluated through a discriminant triangle test (ISO 4120:2004) by comparing the two treatments at each storage time (C0 vs. HP0, C7 vs. HP7, C14 vs. HP14). Seventeen assessors were involved and the significance level was set at 0.01. The pieces of cheeses were served at room temperature using white plastic dishes marked each using a 3-digit code. The tasting station was lighted in order to prevent the perception of differences in colors of the samples. The samples were also described by qualitative descriptive analysis (QDA), according to Stone et al. (22). Attribute terms for evaluation of cheeses were developed by the 17 panelists using QDA methodology. Briefly, ballot development and panelist training were accomplished during seven working sessions. The descriptive terms developed for each major sensory attribute category are reported in **Table 3**. Each attribute was presented as a separate unstructured line scale that recorded panelist responses in increments of 0.1 between 1 (leftmost position) and 15 (rightmost position). The cheese samples were cubed (~1 cm each side) and were presented on white paperboard plates. The panelists also had available an entire transverse slice of each cheese for evaluating appearance attributes. The samples were identified using random 3-digit codes and were at room temperature at the time of testing.

### Extraction and Detection of Odor Active Compounds (OACs)

Qualitative analysis was performed in order to study and compare the aroma profile of C and HP cheeses. Odor active volatile compounds (OACs) extraction was realized by using static headspace solid-phase microextraction (SPME) using a DVB/CAR/PDMS fiber (Supelco, Bellefonte, PA, USA). Ten grams of cheese samples were put into a 22-mL vial and conditioned in a water bath at 40°C for 30 min. Further 40 min of fiber exposition time was required for the static headspace extraction. The fiber was preconditioned before initial use by inserting it into the injector port of a gas chromatography instrument for 1 h at 270°C, and was reconditioned between extractions at the same temperature for 5 min, followed by 10 min at room temperature. Solid phase micro-extraction shows many advances due to the fact that it is a solventless technique and non-artifact forming method as a consequence. It requires a minimal manipulation and small amount of sample (23). Nowadays SPME is commonly used for dairy products flavor extraction such as Camembert cheese (24), Cheddar cheese (25), smear-ripened cheese (26), Trachanas fermented cheese (27), milk proteins (28). Solid phase micro-extraction has been also used for the isolation of odor active compounds of several animal feeds for small ruminants (29).

The detection of the extracted volatile compounds was performed by Gas Chromatography Olfactometry (GC/O) using the single sniff method as described by Marin et al. (30). The OACs were desorbed and separated into a modified Hewlett Packard 6890 GC (Datu, Inc., Geneva, NY, USA) using a fused-silica HP-5 capillary column (30 m × 0.25 mm ID × 0.25 μm film thickness, Agilent Technologies, USA). The chromatographic conditions were as follows: splitless injection at 250°C; oven temperature programme: 35°C for 3 min, 6°C min^−1^ to 190°C, then 30°C min^−1^ to 225°C and 225°C for 3 min; He carrier gas, column flow rate 1.9 mL min^−1^. The eluted compounds were mixed with a stream of humidified air using a method described by Acree and Barnard (31) and the “sniffer” was exposed to this source continuously for 30 min. The response time for each perceived individual odor was recorded by Charmware software (v.1.12, Datu, Inc., Geneva, NY, USA). Retention times (RT) for each OAC were converted into retention indices (RI) and were displayed by the software as a series of peaks in an “aromagram”. RI values were calculated relative to a series of normal alkanes (C7–C18) previously injected into the flame ionization detector (FID) port of the same gas chromatograph. The identification of OACs were carried out by using a GC/Mass Spectrometer (Hewlett Packard 6890), under the same extraction and chromatographic conditions used for GC/O analysis.

This procedure permitted direct comparisons between RI and RT values obtained from GC/O and GC/MS, respectively.

Each odor compound detected by GC/O analysis was defined by a RI value and an odor description. Odor active compounds were also identified using the Flavornet internet database (32), containing over 550 VOCs identified using GC/O techniques. The extraction and detection of flavor profile were performed twice at the same operative conditions in order to confirm odor perception results.

### Smart Nose

Smart Nose is an electronic nose, that allows the direct analysis by MS of volatile organic components from liquid and solid samples without separation of the headspace components.

The analysis was performed with a Smart Nose system incorporating an autosampler CTC Analytics AG (CTC Combi Pal with the Cycle Composer software), a high-sensitivity quadrupole mass spectrometer (Inficon AG) with an ionic mass detection, ranging from 1 to 200 amu, and a user-friendly multivariate analysis software (Smart Nose 1.51) for data acquisition.

Four milliliter of milk were filled into 20-ml vials (adapted for the Combi Pal autosampler).The samples were analyzed in triplicate and randomly placed in the autosampler trays to avoid biases due to external factors.

The main operating conditions were as follow: incubation temperature, 60°C; incubation time, 30; injector temperature, 160°C; purge gas, nitrogen; purge flow, 200 ml/min; Syringe purge time, 1 min. Mass spectrometer scan speed; Mass range, 10–160 amu; speed scan: 2 mass/s; SEM voltage 1540.

## Statistical Analyses

Data on cheese chemical and fatty acid composition were analyzed using a one-way ANOVA to test the effect of the dietary treatment (C vs. HP). Data on cheese sensory characteristics were analyzed using a GLM mixed model to test the effect of the dietary treatment (C vs. HP) and of the time of storage (0, 7, 14), while the variable panelist was considered as random effect. Student's *t*-tests (α = 0.05) were used to determine differences between diet and storage time means when significant differences for that effect were found. SMart Nose data set were transformed using the software supplied with the SMart Nose. First, the mean value of the second and third cycle was calculated. Then the processed data set was normalized using the atomic ion of argon (m/z = 40) from air. This mass to charge ratio is subject to practically no contamination from other compounds and the concentration of this gas in the headspace can be considered as constant. Then a Principal Components Analysis (PCA) was applied. Principle Components Analysis (PCA) is a multivariate statistical analysis that allows to correlate several variables in bi-dimentional plot. In detail, Smart Nose is set in a range of 10–160 amu of ion fragments. PCA allocates a Principal Component (PC) to a single ion fragment and it gives an order from 1 to n ion fragments. PC1 represents the PC with the highest explained variability, the successive (PC2, 3, 4….) represent PC with lower explained variability.

## Results

### Feed Composition

As reported in Table 1, the HP chemical composition is characterized by a high content of crude fat (226 g/kg DM) and fiber fractions (510, 388 and 203 g/kg DM for NDF, ADF and ADL, respectively). As regard fatty acids, HP was characterized by the prevalence of oleic acid (11 g/kg DM), and linoleic acid (2 g/kg DM). Except for the protein content, planned to be similar between the two concentrates, the chemical composition of HP concentrate reflects the composition of the hazelnut peel. In particular, the 36% hazelnut peel inclusion in the HP concentrate increased crude fat (91 vs. 16 g/kg D;), oleic (4.5 vs. 0.3 g/kg DM) and linoleic acid (1,5 vs. 0.9 g/kg DM), and all the fiber fractions in comparison with the C concentrate.

### Cheese Composition

The feeding treatment affected the chemical composition of cheese (Table 2). Specifically, HP cheese was greater in lipid (31.9 vs. 26.5 %; *P* < 0.001) but showed a lower ash content (3.29 vs. 3.76%, *P* < 0.001). As regard lipid profile, the percentage of total saturated (SFA; 56.1 vs. 73.9, *P* < 0.001), polyunsaturated (PUFA; 6.45 vs. 6.81, *P* = 0.007) and odd and branched-chain fatty acids (OBCFA; 3.4 vs. 5.31, *P* < 0.001) were lower in HP cheese as compared to C cheese. On the contrary, a significant increase of monounsaturated fatty acids (MUFA, *P* < 0.001) was found in HP cheeses.

**Table 2:** Chemical and fatty acid composition of cheeses produced with milk of ewes fed with Control (C) or Hazelnut Peels (HP) diets.

**Parameter**	**Diet**		**SEM**	***P*-value**
	**C**	**HP**		
**CHEMICAL COMPOSITION (G/KG DRY MATTER)**				
Moisture	40.02	38.87	1.55	0.272
Fat	26.51^b^	31.94^a^	0.82	<0.001
Ash	3.76^a^	3.29^b^	0.10	<0.001
Protein	25.29	24.26	1.29	0.241
**FATTY ACIDS (G/100G TOTAL FATTY ACIDS)**				
SFA	73.94^a^	56.07^b^	0.36	<0.001
MUFA	15.87^b^	34.48^a^	0.26	<0.001
PUFA	6.81^a^	6.45^b^	0.09	0.007
OBCFA	5.31^a^	3.40^b^	0.04	<0.001

ab *means with superscripts differ significantly (P < 0.05) within that effect*.

### Sensory Analysis

For all times of conservation (0, 7, and 14 days) the triangle test was significant with α = 0.01. At time 0, 13 out of 17 answers were correct, whereas after 7 and 14 days 15 out of 17 were correct. The mean attribute ratings from sensory analysis of Control (C) and Hazelnut Peels (HP) cheeses at different times of storage (0, 7, and 14 days) at 8°C and 80% humidity are reported in Table 3. Diet had a significant effect on holes (*P* < 0.05) and a marginal effect on spicy and acid attribute (*P* < 0.10). In particular, HP cheeses showed fewer holes (4.21 vs. 3.71) and were evaluated less spicy (1.92 vs. 1.35), but were more acid (3.03 vs. 3.70) compared to C cheeses. Time of storage had a significant impact on rim color and oiliness (*P* < 0.05) and a marginal effect on rind thickness (*P* < 0.10). All these attributes decreased with increasing time of storage. The interaction between diet and time of storage effect was not significant.

**Table 3:** Mean attribute ratings and standard error from sensory analysis of Control (C) and Hazelnut Peels (HP) cheeses at different storage times (0, 7, and 14 days) at 8°C and 80% humidity.

**Attribute**	**Diet**			**Storage time (days)**			
	**C**	**HP**	**St err**	**0**	**7**	**14**	**St err**
**APPEARANCE**							
Rind color	3.17	3.35	0.28	3.00	3.12	3.66	0.32
Rind thickness	2.32	2.19	0.25	2.57	2.34	1.85	0.28^*^
Rim color	3.66	3.75	0.27	4.42^a^	3.40^b^	3.29^b^	0.33^**^
Rim thickness	3.20	3.16	0.25	3.52	3.06	2.96	0.29
Color	3.15	3.25	0.28	3.50	2.96	3.14	0.32
Homogeneity	4.89	4.38	0.26	4.69	4.48	4.73	0.32
Holes	4.21^a^	2.71^b^	0.30^**^	3.11	3.70	3.56	0.36
Moistness	3.18	3.63	0.26	3.59	3.46	3.16	0.31
**AROMA**							
Overall intensity	4.94	4.97	0.28	5.09	4.99	4.79	0.33
Fresh milk	4.03	4.51	0.32	4.33	4.41	4.07	0.38
Vegetal	3.11	3.08	0.32	2.80	3.36	3.14	0.37
Floral	2.14	1.81	0.35	1.95	1.93	2.04	0.38
Fruity	2.04	1.69	0.35	1.85	1.64	2.10	0.39
Roasted	2.26	1.86	0.29	2.14	2.00	2.04	0.34
Spicy	1.92	1.35	0.28^*^	1.52	1.66	1.72	0.32
**TASTE**							
Sweet	3.07	2.61	0.28	2.63	3.09	2.80	0.32
Salty	3.25	3.32	0.33	3.59	2.91	3.35	0.38
Acid	3.03	3.70	0.33^*^	3.69	3.34	3.06	0.38
**TEXTURE**							
Consistency	4.27	4.41	0.29	3.95	4.44	4.63	0.34
Homogeneity of consistency	4.77	5.14	0.30	4.49	5.43	4.95	0.35
Chewiness	5.07	5.42	0.31	5.29	5.28	5.16	0.36
Solubility	4.35	4.74	0.35	4.82	4.52	4.29	0.39
Hardness	3.31	2.96	0.30	3.12	3.41	2.89	0.36
Friability	3.14	3.29	0.36	3.06	3.56	3.03	0.42
Adhesiveness	2.43	2.21	0.27	2.52	2.28	2.15	0.32
Graininess	3.08	3.28	0.27	3.40	3.21	2.93	0.33
Elasticity	3.43	3.65	0.31	3.61	3.68	3.33	0.36
Oiliness	4.04	4.30	0.33	4.90^a^	3.84^b^	3.76^b^	0.38^**^
Pungency	2.71	2.78	0.28	3.01	2.69	2.53	0.34
Astringency	1.58	1.92	0.28	2.12	1.66	1.46	0.33
Aftertaste	4.39	4.23	0.31	4.04	4.21	4.68	0.36
Persistence	4.68	5.11	0.30	5.13	4.71	4.85	0.36

ab *means with superscripts differ significantly (P <0.05) within that effect*.

* P <0.10

** *P <0.05*.

### Odor Active Compounds by Gas Cromatography/Olfactometry and Mass Spectrometry

A total of six C and HP cheese batches were analyzed by Gas Cromatography/Olfactometry (GC/O) and GC/Mass spectrometry (GC/MS). In general, C and HP cheese samples showed a poor and very similar volatile profiles (Table 4). No significant difference in number and type of volatile compounds between C and HP groups were found. Some volatile compounds were found in all cheeses. In detail, two esters ethyl butyrate and ethyl octanoate, three ketons diacetyl, 1-octen-3-one and 2-nonanone and one terpene, β-carene were detected as common compounds for both C and HP cheese batches. Moreover, the monitored storage at 0 to 14 days did not affect volatile profile during the shelf life of cheeses. At 7 days of storage, HP cheese showed relevant differences for specific volatile compounds. In detail, HP7 group showed the highest number of esters, two of which were detected only in this sample, and two sulfo-organic compounds also revealed as unique.

**Table 4:** Odor active compounds in control (C) and Hazelnut Peels (HP) cheeses at different storage times (0, 7, and 14 days) at 8°C and 80% humidity extracted by solid-phase microextraction (SPME).

**Compound**	**Chem class**	**Odor perception**	**LRI^**a**^**	**Ident^**b**^**	**C0**	**HP0**	**C7**	**HP7**	**C14**	**HP14**
pentanal	aldehyde	pungent, apple	725	PI,MS		x				x
2-hexenol	alcohol	fruit	881	PI	x					
ethyl butyrate	ester	apple	790	PI,MS	x	x	x	x		x
2-methylbutyl acetate	ester	fruit	891	PI				x		
ethyl isohexanoate	ester	orange	957	PI				x		
ethyl octanoate	ester	wine	1186	PI,MS	x	x	x	x	x	x
diacetyl	ketone	butter	638	PI	x	x	x	x	x	x
1-octen-3-one	ketone	mushroom	970	PI,MS	x	x	x	x	x	x
2-nonanone	ketone	milk	1090	PI,MS	x	x	x		x	x
dimethyl pyrazine	pyrazina	roasted,toasted	911	PI						x
thiophene	sulfur	garlic	665	PI,MS				x		
methylfuranthiol	sulfur	broth	858	PI				x		
3-isothiocyanato-1-propene	sulfur	garlic	886	PI	x				x	
β-carene	terpene	orange	993	PI,MS	x	x	x	x	x	x
**Total**					**8**	**7**	**6**	**9**	**6**	**8**

a *LRI, Linear Retention Index wihHP-5 column*.

b *Identification: PI published Index on flavornet data base (http://www.flavornet.org/flavornet.html); MS, mass spectrometry*.

### Volatile Fingerprint by SMart Nose

**Figure 1:**
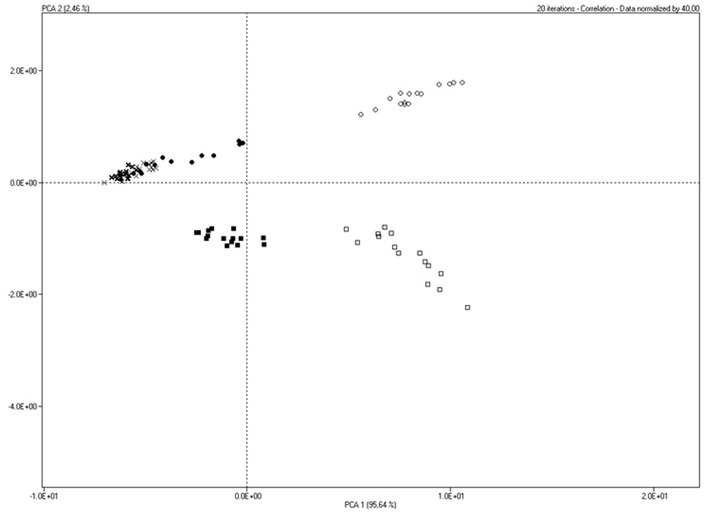
Smart Nose score plot of cheese samples produced with milk from sheeps fed with Control (C) or Hazelnut Peels (HP) diet at different storage time (0, 7, or 14 days) at 8°C and 80% humidity.

Principal Component Analysis from SMart Nose data showed a clear separation among hazelnut peel cheese vs. control cheese at 0 and 7 days (PC1 95,64%; PC2 2,46%). In detail, PC1 separated C0, C7 and C14 from HP0 and HP7. PC1 also explained similar volatile profile of C and HP at 14 days that resulted overlapped in the score plot (Figure 1). Data concerning PC2 highlighted similar volatile profile for C and HP groups at 0 days that were separated from C and HP groups at 7 and 14 days. SMart Nose also revealed major differences for HP7 group than the other cheeses.

## Discussion

### Cheese Chemical Composition

The chemical composition of HP used in the present study is consistent with previous studies describing the nutritive characteristics of hazelnut by-products (5, 33). The animal diet is one of the most important factors affecting the chemical composition of ruminant products. In particular, it is known that providing a diet reach in fat usually increases the fat percentage in milk and cheese (34). Therefore, not surprisingly HP cheese showed a greater percentage of fat in comparison with C cheese. Similarly, the fatty acid composition of ruminant derived products can be partially manipulated by the diet (8). Therefore, the greater percentage of MUFA found in the HP cheese and the contemporary reduction of SFA could be due to the fatty acid composition of the concentrate supplied to HP ewes. In human, the consumption of MUFA is positively associated with the reduction of both plasma LDL cholesterol and triacylglycerol concentrations, thus reducing the risk of cardiovascular diseases (35). Moreover, HP cheese had a lower SFA to UFA ratio in HP cheese. Literature reports that the intake of unsaturated fatty acid should be increased at expense of SFA. Therefore, the administration of dietary improved the nutritive value of the HP cheese.

### Sensory Profile

The triangle test used as a discriminant tool to assess whether trained assessors were able to distinguish between the 2 differently treated samples confirmed that the HP and C cheeses' overall sensory profiles were significantly different one each other, because, 13 and 15 persons, after 7 and 14 days of storage, respectively, correctly answered to the test, hence, a number higher than the minimum one (11) necessary to conclude that significant differences existed between the two samples compared, according to the ISO 4120:2004, and the α risk selected (0.01). Regarding the QDA results, the statistical analysis of the attribute ratings collected from 17 trained panelists evidenced a significant reduction of holes and spiciness and an increase of acidity in HP compared to C cheeses. These results agree with the study reported in Torri et al. (36) based on consumers' acceptability of cheeses enriched with 0.8, 1.6, and 2.4% of grape skin powder from 2 different vines (Barbera and Chardonnay), added during cheese-making. The addition of grape skin powder highly influenced sensory properties of such innovative products, above all in terms of appearance and texture. White color, homogeneity and elasticity of the paste and the presence of lactic odor positively influenced consumers' preference. Conversely, the appearance of marbling, granularity, sandyness, acidity, saltiness, and astringency negatively affected the acceptability of the cheese when the quantity of added grape skin powder exceeded 0.8 and 1.6% for Barbera and Chardonnay, respectively. However, the higher intensity of acidity found in this study for HP cheeses was marginal. Presumably the higher presence of holes mostly determined the significance of the triangle test. Contrasting results, on the contrary, were obtained by several authors about the effect of using milk obtained from cows or goats fed with flax extruded, on possible off-flavors products due to lipid oxidation.

Dubreouq et al. (37) and Gaborit et al. (38) have shown how the addition of flax extruded in the diets of dairy cows and goats not integrated with antioxidants, besides improving the acidic composition of the lipid fraction of the cheese, important for a health point of view, determined the production of off-flavors (metallic and oxidized flavor). The production of off-flavors was not observed by Lerch et al. (39) in aged Saint-Nectaire cheese. However, according to the authors themselves, the problem could arise for long-ripened cheeses in which an important role is played by lipolysis levels that could increase susceptibility to the oxidation of PUFAs. The cheeses examined in this study were sampled right after aging for 40 days, thus they can be considered fresh cheeses. Further investigation would help to understand the effect of the inclusion of polyphenols extracts in longer maturation periods.

### ODOR Active Compounds and Volatile Fingerprint by GC/O and SMart Nose

The volatile profile of C and HP cheese samples showed a good similarity. These results could be explained by the short ripening time (time 0 = 48 days). Cheese ripening is a complex process in which three flavor generating pathways, glycolysis, lipolysis and proteolysis, are initially involved, leading to secondary metabolites production responsible for flavor precursors origin (40). As a consequence, ripening time plays an important role in flavor development (41).

Looking into every single cheese batch, in C group, 2-hexenol was detected only in C0, degrading in C7 and C14. The alcohol, moreover detected as unique compound among all cheeses, was likely processed to secondary products. Two ester compounds were found in C groups: ethyl butyrate was detected in C0 and C7, whereas ethyl octanoate was found in all C cheese samples. Three ketones (diacetyl, 1-octen-3-one and 2-nonanone) were also detected as common in all C group. A sulfur compound, 3-isothiocyanato-1-propene, was detected at 0 and 14 days of storage but not at 7 days.

In HP group, HP0 and HP14 showed the same profile, except for dimethyl pyrazine, detected as unique compounds in HP14. Pyrazine could derive from the hazelnut feeding treatment.

The esters form through free fatty acids and alcohols reaction, giving an important impact to cheese flavor profile (42–46). In this study, ester was detected as the main representative chemical class, conferring fruity notes to the cheeses.

Two sulfur compounds, thiophene and methyl furanthiol, were identified as unique in HP7 cheese group. In general, sulfur compounds originate from the catabolism of sulfur-amino acids (42). Beside the well-known antimicrobial activity (47), recent studies on antioxidant protection grade of cheese report an increasing antioxidant effect in cheese during ripening time in parallel with thiophene and methyl furanthiol production (48, 49). The unique aldehyde extracted by SPME was detected only in HP0 and HP14 cheeses. Moreover, 2-nonanone was found in all cheeses except for HP7. The aldehyde and 2-nonanone are considered fat oxidation products. The absence of these volatile compounds in HP7 suggests a higher antioxidant protection grade of this cheese than the others.

Further studies on vitamin content should be conducted in order to investigate the interactions between the presence of antioxidant volatile compounds and the oxidative stability of ewe cheese by calculating the Degree of Antioxidant Protection (DAP).

Results from SMart Nose were confirmed by GC/O analysis. SMart Nose score plot (Figure 1) showed a clear separation of HP7 group from the others, suggesting a different volatile profile. The separation of HP7 group was supported by GC/O results, in fact, in HP7 cheeses, two unique esters (2-methylbutyl acetate and ethyl isohexanoate) and two unique sulfur compounds (thiophene and ethylfuranthiol) were detected, whereas, pentanal and 2-nonanone, this latter revealed in all the other cheeses, were absent. The olfactive responses could be responsible of HP7 behavior in the SMart Nose score plot.

## Conclusions

The experiment presented in this paper aimed at the evaluation of the inclusion of hazelnut peels in the diet of dairy ewes on the chemical and sensory characteristics of cheeses. Sensory analysis revealed a clear distinction between the 2 experimental groups, whereas the sensory profile showed a marginal production of off-flavors linked to spicy and acid attributes for the cheeses produced with milk of ewes fed with the addition of hazelnut peels. However, lipolysis levels in longer ripening period could increase susceptibility to the oxidation of PUFAs, therefore further investigation would help to understand the effect of the inclusion of polyphenols extracts in longer maturation periods. The volatile profile showed the absence of some volatile compounds in HP cheeses, suggesting a higher antioxidant protection grade linked to the inclusion of HP in the diet. Further studies on vitamin content should be conducted in order to investigate the interactions between the presence of antioxidant volatile compounds and the oxidative stability of ewe cheese.

## Author Contributions

GL and MP designed and conducted the experiment. BV and AP carried out fatty acid composition analysis and mainly contributed to the conception and structure of this manuscript. VM interpreted results from the antioxidant point of view. GB contributed reagents, materials, tools, and interpretation of results for SmartNose analysis. TR detected odor active compounds and contributed to the manuscript for the aroma and SmartNose results and discussion. SE, AT, and MS performed sensory analysis. MC analyzed, interpreted, and discussed sensory analysis data and coordinated and complemented authors' contributions to the manuscript. All authors have read and approved the final manuscript.

### Conflict of Interest Statement

The authors declare that the research was conducted in the absence of any commercial or financial relationships that could be construed as a potential conflict of interest.
